# Balancing Benefits and Risks: A Literature Review on Hypersensitivity Reactions to Human G-CSF (Granulocyte Colony-Stimulating Factor)

**DOI:** 10.3390/ijms25094807

**Published:** 2024-04-28

**Authors:** Roxana Silvia Bumbăcea, Mihaela Ruxandra Udrea, Selda Ali, Violeta Claudia Bojincă

**Affiliations:** 1 Allergology Department, “Carol Davila” University of Medicine and Pharmacy, 050474 Bucharest, Romania; roxana.bumbacea@umfcd.ro (R.S.B.); selda.ali@umfcd.ro (S.A.); 2 Department of Allergology and Clinical Immunology, “Carol Davila” Nephrology Clinical Hospital, 010731 Bucharest, Romania; 3 Clinical Department 5, “Carol Davila” University of Medicine and Pharmacy, 050474 Bucharest, Romania; violeta.bojinca@umfcd.ro; 4 Department of Internal Medicine and Rheumatology, “Sfânta Maria” Hospital, 011172 Bucharest, Romania

**Keywords:** G-CSF/granulocyte colony-stimulating factor, filgrastim, lenograstim, hypersensitivity, allergy, anaphylaxis, drug hypersensitivity reactions

## Abstract

Human granulocyte colony-stimulating factor (G-CSF) is a granulopoietic growth factor used in the treatment of neutropenia following chemotherapy, myeloablative treatment, or healthy donors preparing for allogeneic transplantation. Few hypersensitivity reactions (HRs) have been reported, and its true prevalence is unknown. We aimed to systematically characterize G-CSF-induced HRs while including a comprehensive list of adverse reactions. We reviewed articles published before January 2024 by searching in the PubMed, Embase, Cochrane Library, and Web of Science databases using a combination of the keywords listed, selected the ones needed, and extracted relevant data. The search resulted in 68 entries, 17 relevant to our study and 7 others found from manually searching bibliographic sources. A total of 40 cases of G-CSF-induced HR were described and classified as immediate (29) or delayed (11). Immediate ones were mostly caused by filgrastim (13 minimum), with at least 9 being grade 5 on the WAO anaphylaxis scale. Delayed reactions were mostly maculopapular exanthemas and allowed for the continuation of G-CSF. Reactions after first exposure frequently appeared and were present in at least 11 of the 40 cases. Only five desensitization protocols have been found concerning the topic at hand in the analyzed data. We believe this study brings to light the research interest in this topic that could benefit from further exploration, and propose regular updating to include the most recently published evidence.

## 1. General Data on G-CSF

### 1.1. Biological Function

Human granulocyte colony-stimulating factor (G-CSF), also known as colony-stimulating factor 3 (CSF 3), is a 19.6 kDa glycoprotein structurally composed of 175 amino acid residues [1]. This granulopoietic growth factor is naturally synthesized by multiple immune cells within the human body and functions as both a cytokine and hormone. Primarily, G-CSF serves the critical role of stimulating the production of granulocytes and stem cells from the bone marrow, facilitating their subsequent release into the bloodstream.

G-CSF exhibits additional influence on monocytes, lymphocytes, and the hemostatic system [2]. Notably, it not only increases the number of peripheral blood monocytes but also enhances circulating eosinophil levels and adhesion [3,4].

Another pivotal role of G-CSF is in fostering the survival, proliferation rate, differentiation, and function of neutrophil precursors. Upon binding to receptors, it triggers maturation through the activation of Jak/STAT (Janus kinase/signal transducer and activator of transcription) and MAPK (mitogen-activated protein kinases) pathways [5].

Beyond these functions, ongoing research explores its association with neurogenesis and angiogenesis, showcasing implications in cancer progression [6] and potential contributions to brain regeneration after injury [7].

This multifaceted understanding of G-CSF underscores its diverse and significant roles, as well as its potential therapeutic applications.

### 1.2. Medical Use

G-CSF, along with other hematopoietic growth factors such as granulocyte–macrophage colony-stimulating factor (GM-CSF), interleukins (IL-1, IL-3, IL-4, and IL-6), macrophage colony-stimulating factor (M-CSF), epoetin (erythropoietin), and stem cell factors (SCFs), plays a crucial role in regulating the initial stages of hematopoiesis. These factors have proven to be valuable in treating cytopenia following chemotherapy and was first used in 1988.

G-CSF serves as a mobilizing factor for peripheral blood progenitors before myeloablative treatment, either with autologous bone marrow transplantation or in donors for allogeneic transplantation. Additionally, it aids in mobilizing granulocytes for transfusions and contributes to the treatment of congenital or acquired bone marrow failure [8,9].

Furthermore, G-CSF administration has shown efficacy in patients experiencing non-chemotherapy idiosyncratic drug-induced neutropenia, commonly associated with rheumatic or psychiatric drugs or even disease-induced neutropenia. This treatment has resulted in a shortened duration of mentioned cytopenia, antibiotic therapy, and hospitalization [10].

G-CSF is also explored as a therapeutic option to extend survival in advanced HIV infection or for patients with a history of severe, repeated infections [11].

The frequency of use remains largely unknown and is dependent on various factors, such as the incidence of neutropenia and its severity and the likelihood of febrile neutropenia, which are all closely linked to the chemotherapy regimen employed. The type of malignancy and patient-specific characteristics (gender, age, presence of concomitant disease, and general health status) all pose a challenge in estimating the frequency of G-CSF usage. Current guidelines advocate for primary prophylaxis with G-CSF during the first cycle of chemotherapy, and subsequent ones when the risk of febrile neutropenia exceeds 20% [12].

The posology of G-CSF varies depending on the therapeutic indications, ranging from 5 µg/kg/day 24 h after chemotherapy to 10 µg/kg/day 24 h after myeloablative treatment or bone transplantation. Generally well-tolerated, G-CSF can be administered via a subcutaneous or intravenous route.

### 1.3. Pharmaceutical Variants

Exogenous production of G-CSF is based on the recombinant DNA technology of bacterial, yeast, or mammalian cells.

Two types of recombinant human granulocyte colony-stimulating factors are currently available. The Escherichia coli-derived G-CSF, known as filgrastim, is a non-glycosylated molecule that acts similarly to endogenous G-CSF. Lenograstim is a glycosylated natural product formed from Chinese hamster ovary cells.

Over time, biosimilars, biological medicinal products, have received marketing authorization, and the agents have both similar safety profiles [13] as well as quality and efficacy characteristics similar to the originator. Biosimilar G-CSFs are complex, micro-heterogeneous proteins from a structural point of view, manufactured from genetically modified living cells via multiple purification and formulation methods. Several such molecules have been approved, all of which have the same aforementioned therapeutic indications. Long-acting G-CSFs (L-G-CSFs) are the PEGylated forms of short-acting G-CSFs (S-G-CSFs), with augmented half-lives in serum after subcutaneous administration.

Nowadays, besides filgrastim and lenograstim, eight major G-CSF drugs are known: long-acting filgrastim—pegfilgrastim, lipegfilgrastim, mecapefilgrastim [14], empegfilgrastim [15], S/L-G-CSF biosimilar—balugrastim [16], leridistim, pegleridistim [17], and pegteograstim [18].

### 1.4. Adverse Reactions to G-CSF

When a recombinant protein, in this case G-CSF, is strikingly similar to the endogenously produced one, it is able to induce a production of antibodies. As a result, either there is no noticeable effect, or the antibodies neutralize the endogenous protein, resulting in multiple side effects.

The administration of G-CSF is associated with a range of adverse effects, among which general musculoskeletal pain is prevalent, affecting approximately 20 to 25% of patients [19]. The term encompasses not only bone pain, but also arthralgia, myalgia, jaw, and extremities pain. Additional common side effects include headache, fatigue, and nausea.

Splenomegaly has also been noted, with some patients (25%) experiencing asymptomatic increases in splenic volume, with the sole indicator often being abdominal or shoulder tip pain [13]. In severe chronic neutropenia cases [20,21], splenomegaly with splenic extramedullary hemopoiesis of all three lineages has been described [20], particularly in patients with refractory disease.

Felty’s syndrome, a condition characterized by rheumatoid arthritis, granulocytopenia, and splenomegaly, has shown varied responses to G-CSF treatment. Several cases have been published regarding patients with reactivation or worsening symptoms, such as joint pain and swelling and an increase in acute phase proteins [21,22,23], potentially induced by direct neutrophil activation and migration into the joints. Despite these reactions, the usefulness of G-CSF was proven and this treatment has also been used for patients with hyperimmunoglobulin M syndrome [24] and Sjogren’s syndrome [25].

Effects on the cardiovascular system are also a concern, with reports suggesting fatal vascular events like arrhythmias, coronary thrombosis, and arterial thrombosis, possibly linked to a hypercoagulable state induced by G-CSF [26,27,28,29,30,31], as well as cases of aortitis [32,33,34,35,36] or large-vessel vasculitis (LVV) [37].

Other possible side effects refer to pulmonary involvement ranging from respiratory failure due [38] to interstitial pneumonia [39,40,41,42,43,44] (mostly due to increased toxicity of other drugs), or hepatic [45,46,47], renal [19,48,49] (with transient hematuria [50] during long-term treatment), and hematologic symptoms [51,52,53,54,55,56,57]. 

From a dermatological standpoint, G-CSF therapy has been linked to various manifestations, such as pyoderma gangrenosum [58], Sweet’s syndrome [59,60,61,62,63,64,65], other neutrophilic dermatoses [66], granulomatous dermatitis [67], widespread folliculitis [68], cutaneous vasculitis [50,69,70,71,72], and exacerbations of acne [73] and psoriasis [70,74,75]. Several cases of cutaneous eruptions containing leukemic cells have been reported (without their presence in the bone marrow or blood) after G-CSF, suggesting the skin’s ability to simulate malignancies, as well as its dependency on G-CSF administration [76,77].

These adverse effects highlight the importance of careful monitoring and risk assessment when utilizing G-CSF in clinical settings. Moreover, attention must be given to drug hypersensitivity reactions (DHRs), a group of adverse effects that may require careful monitoring given their potentially life-threatening nature and which may impose targeted therapeutic interventions.

## 2. Hypersensitivity Reactions to G-CSF

### 2.1. Overview

Hypersensitivity reactions (HRs) represent an exaggerated immune response to the administration of a drug. In some individuals, the introduction of exogenous G-CSF may trigger an immune response characterized by an abnormal sensitivity. This hypersensitivity can manifest as a spectrum of reactions, ranging from mild symptoms such as rash to more severe manifestations like anaphylaxis. The mechanisms underlying hypersensitivity to G-CSF are multifaceted and may involve immunoglobulin E (IgE)-mediated pathways, immune complex formation, or other immune system components. Understanding the intricacies of hypersensitivity reactions to G-CSF is essential for optimizing the safety and efficacy of G-CSF therapy.

Growth factors have been rarely associated with hypersensitivity reactions. In 1988, a phase III study [78] (multicenter, randomized, and double-blind-placebo-controlled) included 211 patients treated for small-cell lung cancer. They were randomized to receive either filgrastim or placebo. The main purpose was to determine the safety of the drug and whether adverse effects and hypersensitivity reactions could be attributed to the administration of G-CSF. Mild generalized skin rash or itching were observed in about 6% of patients in both groups (G-CSF and placebo), and thus it could not be assertively concluded that the causative agent was G-CSF.

Later on, Bustillo et al. estimated the incidence of rash to be less than 3.7% in patients receiving the PEGylated form of G-CSF [79]. Nowadays, few mentions of mild cutaneous reactions can be found in the literature, such as local injection-site reactions [80], isolated pruritus, or rash, most likely being underreported due to their non-life-threatening nature.

There are currently no existing data on the prevalence of more severe hypersensitivity reactions, with only a small number of cases having been published in the literature in the last 30 years. To establish the frequency of these reactions and help clinicians balance the benefits and risks of G-CSF administration, we performed a literature analysis of hypersensitivity reactions.

### 2.2. Methods

**Figure 1:**
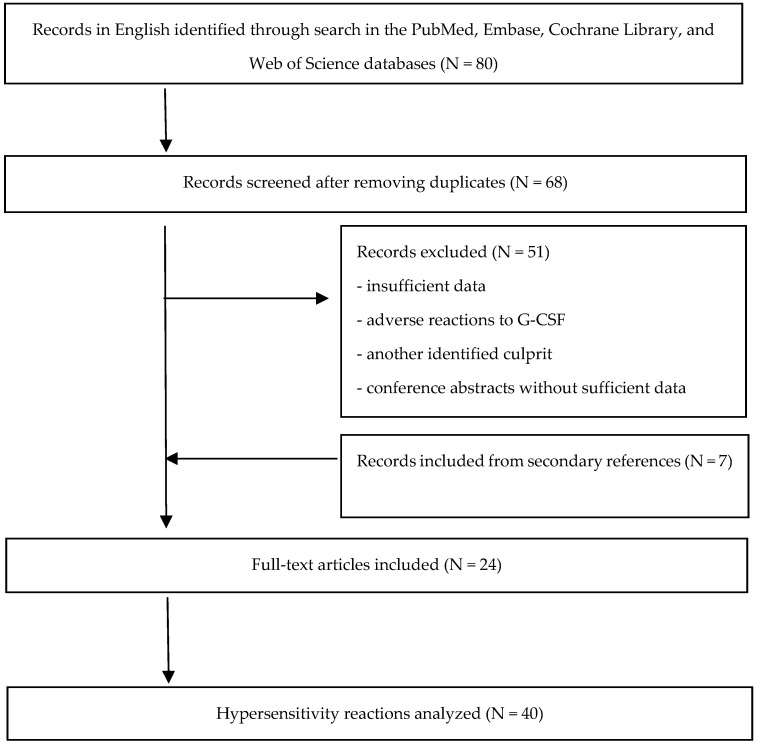
Flowchart of the literature search process.

We reviewed the relevant articles published in English from the time of their market introduction (1988) to January 2024 after a thorough search in the PubMed, Embase, Cochrane Library, and Web of Science databases. The Medical Subject Heading (MeSH) and keywords were used, including “G-CSF/granulocyte colony-stimulating factor”, “filgrastim”, “lenograstim”, “hypersensitivity”, “allergy”, “anaphylaxis”, and “drug hypersensitivity reactions”. This approach was also combined with a manual inspection of references in all selected studies. The search generated 68 entries from case reports, case series, observational studies, and even clinical trials. After removing duplicates, 17 entries were relevant to our study (specifically addressing hypersensitivity reactions to G-CSF, both immediate and delayed ones) and 7 others were found from bibliographic sources, and thereby included in this overview. This resulted in 40 cases of hypersensitivity reaction after G-CSF administration (Figure 1).

The following studies were excluded: (1) reports with insufficient details on hypersensitivity reactions—no clinical presentation, timing, severity and relevant diagnostic tests, (2) reports which misinterpreted the reactions—further classified as adverse, (3) conference abstracts without full-text articles which lacked relevant information for thorough analysis, (4) reports with non-relevant endpoints—primary focus not on hypersensitivity reactions to G-CSF or another causative agent was subsequently identified as the culprit, and (5) reports inaccessible at the moment of writing this review.

Data were extracted from the search characteristics and the most important features were included in Table 1, such as the culprit drug and administration method, the existence of previous administration, and patients’ backgrounds. Moreover, we have considered it of utmost importance to mention the delay of appearance which has allowed for a categorization of hypersensitivity reactions based on the timing of their onset, distinguishing between immediate and delayed manifestations. Immediate reactions were considered to typically occur shortly after G-CSF administration, within minutes to a few hours (<6 h) (more frequently in the first hour after administration). In contrast, delayed reactions manifested over a more extended timeframe following drug administration (>6 h to days). The decision for using the cutoff of 6 h stems from the classification systems prevalent at the time of earlier studies (the 1-hour cutoff has been proposed in the more recent literature) to maintain consistency throughout the study.

The WAO anaphylaxis scale has emerged as an important consideration in the context of anaphylactic responses [81], guiding us to categorize the reactions according to their severity, from grade 1 anaphylaxis, encapsulating instances characterized solely by cutaneous findings, to grade 5 anaphylaxis, characterized by severe manifestations including hypotension and/or temporary loss of consciousness.

Lastly, we considered it necessary to mention the allergy work-up procedures conducted in each case, as these investigations played a pivotal role in unraveling the underlying immunological mechanisms and guiding the subsequent management of cases.

**Table 1:** Reported hypersensitivity reactions related to G-CSF therapy.

Authors	G-CSF	Route	Background	Severity Grade WAO [57]	Onset after Adm.	No. of Adm.	Allergy Workup SPT/IDT/Others	Management Decision
Jaiyesimi et al. [82] (1991) (2 cases)	F	sc	Felty sdr. (Neutropenia)	A2	300 min	1st	/	UKN
	F	sc	CML	A3	10 min	3rd	/	UKN
2. Sasaki et al. [83] (1994) (2 cases)	F	sc	LC	A1	min	3rd	Positive F, Negative L	Adm. (L)
	L	sc	LC	A1	min	6th	Positive F, Positive L, Total IgE increase	STOP
3. Batel-Copel et al. [84] (1995)	F	iv	ADK	A5	5 min	1st	/	STOP
4. Munoz et al. [85] (1996)	F	sc	SLE (neutropenia)	A1	min	3rd	Positive F	STOP
5. Sullivan and Nelson [86] (1997)	F	sc	AIDS	A1	60 min	14th	/	Adm. (L)
6. Adkins [87] (1998)	F	UKN	HD	A5	50 min	1st	/	STOP
7. Keung et al. [88] (1999)	UKN	iv	BC	A5	min	1st	/	Adm. GM-CSF
8. Khoury et al. [89] (2000) (10 cases)	UKN	sc	CML (10pts)	A1 to A5	A median of 30 min	1st or 2nd	/	STOP (6 pts), Adm. UKN (4 pts)
9. Hanna et al. [90] (2008)	PegF	UKN	BC	A5	10 min	1st	/	STOP
10. Tulpule et al. [91] (2009)	L	sc	AD	A5	40 min	1st	/	STOP
11. Tholpady et al. [92] (2013)	F	sc	HD	A5	90 min	1st	Normal ST	STOP
12. Hronek et al. [93] (2014)	F	sc	MM	A3	2–3 min	2nd	/	Des.F
13. Nunez-Acevedo et al. [94] (2015)	F	sc	BC	A5	5 min	1st	Negative F, L Normal BST	Des.L
14. Amaral et al. [95] (2016)	F	sc	BC	A3	10 min	4th	/	Des.F
15. Yamamoto et al. [96] (2016)	L	sc	HD	A3	60 min	1st	/	STOP
16. Doval et al. [97] (2019)	F	sc	HD	A5	45 min	1st	/	STOP
17. Gonzalez-Cavero et al. [98] (2019)	F + L	UKN	V-EST	A2	240 min	3rd	Negative F	Des.F
18. Jeter et al. [99] (2021)	PegF	UKN	HL	A3	60 min	UKN	Normal BST and c-KIT	Des.F
19. Alvarez-Ruiz et al. [100] (2003)	UKN	sc	CML	Delayed reaction	8 days	1st	/	+++ Adm. Then STOP
20. Alvarez Ruiz et al. [101] (2004) (6 cases)	UKN	UKN	BC	Delayed reaction	2 days	UKN	Biopsy—enlarged, plump macrophages	Adm.
	UKN	UKN	CML	Delayed reaction	13 days	UKN	Biopsy—enlarged, plump macrophages	Adm.
	UKN	UKN	ALL	Delayed reaction	2 days	UKN	Biopsy—enlarged, plump macrophages	Adm.
	UKN	UKN	CML	Delayed reaction	8 days	UKN	Biopsy—enlarged, plump macrophages, elastic fiber phagocytosis	Adm.
	UKN	UKN	NHL	Delayed reaction	24 days	UKN	Biopsy—enlarged, plump macrophages	Adm.
	UKN	UKN	CML	Delayed reaction	3 days	UKN	Biopsy—enlarged, plump macrophages	Adm.
21. Brumit et al. [102] (2003)	UKN	UKN	HD	Delayed reaction	1 day	1st	/	+++ Adm. then STOP
22. Bustillo et al. [79] (2009)	PegF	sc	PC	Delayed reaction	1 day	1st	/	+++ Adm. then STOP
23. Scott et al. [103] (2009)	PegF	UKN	HL	Delayed reaction	1 day	+++	Biopsy—lichenoid drug eruption	STOP
24. Daldla et al. [104] (2014)	PegF	sc	BC	Delayed reaction	9 days	3rd	Biopsy—allergic reaction	STOP

UKN—unknown, F—filgrastim, L—lenograstim, PegF—pegfilgrastim, sc—subcutaneous, iv—intravenous, sdr.—syndrome, CML—chronic myelogenous leukemia, LC—lung cancer, ADK—adenocarcinoma, SLE—systemic erythematous lupus, AIDS—acquired immunodeficiency syndrome, HD—healthy donor, BC—breast carcinoma, AD—asthmatic donor, MM—multiple myeloma, PC—pancreatic cancer, HL—Hodgkin’s lymphoma, V-EST—vaginal endodermal sinus tumor, ALL—acute lymphocytic leukemia, NHL—non-Hodgkin’s lymphoma, A—anaphylaxis, +++—several administrations, SPT—skin prick test, IDT—intradermal test, ST—serum tryptase, BST—baseline serum tryptase, Adm.—administration, pts—patients, des.—desensitization.

## 3. Results and Discussion

After conducting a thorough analysis of all cases, it can be concluded that filgrastim was the most frequently administered G-CSF, with at least 13 of the 40 patients (32.5%) receiving it either intravenously or subcutaneously. Considering the lack of information regarding the administered molecules in some of the cases published [88,89,100,101,102], it is reasonable to assume that this percentage could potentially increase, likely due to the accessibility of filgrastim on the international market. PEG-filgrastim was also one of the preferred molecules, possibly because by “PEGylation” the drug obtains an extended half-life of 15–80 h and a longer effect in the organism because of slower renal clearance.

The underlying pathologies of patients requiring this treatment predominantly involved neoplasms with various localizations. However, healthy donors, as well as patients with systemic erythematous lupus (SLE), Felty syndrome, and acquired immunodeficiency syndrome (AIDS) also experienced reactions induced by G-CSF.

Upon analyzing the time delay after which the reactions occurred, we categorized them into 29 immediate reactions and 11 delayed ones, without being able to clearly specify the underlying mechanism involved.

### 3.1. Immediate Reactions

Immediate hypersensitivity reactions varied in delay, ranging from a few minutes to several hours after drug administration, manifesting after approximately 50 min on average.

The severity of immediate reactions spanned from grade 1 anaphylaxis (only cutaneous implications) on the WAO scale to grade 5 anaphylaxis (resulting in loss of consciousness, hypotension, etc.), with 9 out of 29 reaching the highest degree of severity. In these cases, filgrastim was the most common culprit, in five out of nine cases.

Only two immediate hypersensitivity reactions to pegfilgrastim have been reported. One case [90] is worth detailing: The patient developed anaphylactic symptoms (generalized urticaria, dyspnoea, nausea, angioedema, vomiting, hypotension, and hypoxia) 10 min after the administration of G-CSF. Despite correct treatment, the patient required several doses of epinephrine. Furthermore, in the course of the next week, antihistamines and systemic corticoids were prescribed because of persistent urticaria, facial edema, and recurrent episodes of mild dyspnea. This could be explained by the pharmacodynamics of the drug, as PEGylation changes the structure of the molecule, slowing the renal clearance and leading to persistent plasma levels that may have led to prolonged anaphylactic symptomatology.

There are several instances in which the reaction appeared after multiple administrations of G-CSF, suggesting that sensitization was necessary for the development of hypersensitivity reactions. Regardless, at least 11 reactions occurred at the initial contact with the drug, as mentioned by the authors. This led to implications regarding the mechanism triggering the hypersensitivity reaction, leaving room for suspicion of non-IgE-mediated cases appearing at first exposure to the drug. Another theory suggests that contained excipients are responsible for hypersensitivity reactions. For example, mannitol, known to be in G-CSF [105], could be the culprit. Furthermore, polysorbate 80, contained in filgrastim [106,107], could be the cause of acute urticaria and delayed hypersensitivity reactions. Previous exposure to excipients, leading to sensitization, may therefore explain reactions occurring at the first administration of the drug.

It is also necessary to mention the fact that in at least two cases [82] the causality relationship between the administration of G-CSF and the presence of the hypersensitivity reaction was uncertain, due to the concomitant medication of patients as well as their intricated diseases. The possibility of liaison is supported only by the timing of the reaction.

When it comes to the evaluation of the immediate hypersensitivity reactions, it is notable that allergy skin testing was conducted in only a limited subset of cases, specifically 5 out of the 29 reactions reviewed. Skin testing for G-CSF consisted of a skin prick test followed by an intradermal test. Four out of five tested cases were either grade 1 or 2 anaphylaxis, with only one case reaching grade 5. Interestingly enough, only the grade 1 reactions (three cases) had positive skin tests for the culprit, one being also positive for the alternative. A negative test in severe reactions (grade 5 anaphylaxis) may raise suspicion regarding the mechanism involved in the HR, suggesting a non-IgE mediated one.

Despite the concentration for testing these molecules being known, as shown in Table 2, the scarcity of documented reactions may have impeded the ability to draw definitive conclusions regarding the safety of skin testing, which may explain the lack thereof in the rest of the cases.

Furthermore, it is worth noting the existence of alternative methods of evaluation of hypersensitivity reactions mentioned in Table 1, such as baseline serum tryptase [92,94,99], total IgE [83], and c-KIT [99], but they may have limited impact on further therapeutic approaches given their lack of demonstrated usefulness in these specific contexts. 

All these assessment techniques had profound implications for case management decisions. In instances where allergy work-up was lacking, specifically in reactions where the severity surpassed grade 2, the discontinuation of G-CSF, either as the culprit or a similar molecule, was the preferred route of action in 15 of the 29 cases. The positivity of skin testing, regardless of the severity of the reaction (all were grade 1), prompted the decision to cease G-CSF administration.

Our analysis showed that the possibility of cross-reactivity was also considered before interruption or changing of medication, as one article [83] mentioned the decision to test both filgrastim, the culprit, and lenograstim, an alternative. Skin testing for filgrastim was positive while lenograstim was negative, suggesting that small structural differences do exist, and administration is possible under careful observation. Nevertheless, the concern of cross-sensitivity between polypeptides produced in similar recombinant models remains a topic of interest [105].

Because G-CSF administration is vital for some patients, desensitization protocols have been proposed and successfully applied in five cases, enabling a safe continuation of treatment. Intriguingly, only two out of these five patients were evaluated by skin testing beforehand, and both had negative results. In cases consisting of grade 2 or 3 anaphylaxis, the desensitization was performed using the molecule that caused the initial reaction (three out of five) and led to tolerability without adverse events. Others decided on performing desensitization with an alternative drug (two out of five); in one of these cases, the severity of the reaction (grade 5) may have led to the decision to change molecules. These five protocols are the sole entities found while conducting this study; all available information is synthesized in Table 3. The most used intravenous desensitization protocols were based on an 8- to 12-step approach, with the possibility of adjustment of the target dose and time intervals between doses, whereas the ones that used subcutaneous administration ranged from 12 to 15 steps.

### 3.2. Delayed Reactions

In our research, a limited number of delayed hypersensitivity reactions were identified, totaling 11 cases. Three of these reactions were induced by pegfilgrastim, while for the others the culprit was unknown and referred to only as “G-CSF”. The onset of symptoms exhibited a temporal range from 1 to 13 days following administration, and the morphological characteristics were mostly those of maculopapular exanthemas (8 out of 11) [79,100,101,102].

One case [101] had an atypical evolution of the reaction 24 days after the cessation of G-CSF, but only 5 days after the patient’s chemotherapy treatment. This suggested the existence of another possible culprit and questioned the causal role of G-CSF, without the possibility of exclusion, as no allergy work-up was conducted.

Notably, a minimum of two instances occurred after multiple administrations of the drug, underscoring the importance of repeated drug exposure in the development of delayed reactions. Sadly, most of the cases lacked information regarding the exact number of doses of G-CSF before the onset of the reaction.

Intriguingly, three patients [79,100,102] developed the reaction after the first drug administration but lacked formal evaluation. The characteristics of the reaction, being of non-severe nature, allowed for continued drug administration until the reappearance of the reaction. Six out of the eight reactions described as maculopapular exanthemas benefited from bioptic analysis, showcasing dermal infiltration of enlarged macrophages, vacuolar dermatitis, and spongiosis. Despite the continuous nature of the exanthema, lasting on average 7 days, the G-CSF administration was successfully continued, whether by diminishing the dose or by concomitant usage of corticosteroid therapy (topical or systemic).

In contrast, two cases [103,104] necessitated a biopsy because of the severity of the eruption, revealing histopathological patterns suggestive of drug-induced reactions without further explanation. The extended resolution of these reactions (10 days and 3 weeks, respectively), led to considerations of severity, imposing a cautious approach. The decision was made to refrain from re-administering the drug to mitigate potential risks associated with recurrence.

## 4. Limitations of the Study

The limitations of this study primarily revolve around the potential of missing relevant data. The decision to include only articles published in English may have resulted in the exclusion of valuable information published in other languages. Furthermore, despite our efforts to access a broad range of resources, the search was confined to specific databases, which may have overlooked studies published elsewhere or in gray literature sources.

Another limitation arises in proposing a clear classification regarding the underlying mechanisms involved in the onset of the reactions, as most cases lacked allergy work-ups. Given that an IgE-mediated mechanism was not always identified when analyzing immediate reactions, speculation on other potential mechanisms becomes necessary. Although immediate in nature, these processes may require longer periods for manifestation, due to a pharmacological mechanism. As such, we opted for the 6-hour threshold between immediate and delayed reactions, wanting to uphold consistency over time. It is important to note that this choice may not align with some of the current systematization methods. Additionally, interpersonal variations in metabolism and clearance rates, particularly in individuals classified as poor or extensive metabolizers, could contribute to the occurrence of reactions within this extended timeframe.

## 5. Conclusions

G-CSF is considered a relatively safe treatment that can rarely cause hypersensitivity reactions, ranging from low-risk, cutaneous eruptions to anaphylaxis and even delayed reactions mostly defined as maculopapular exanthemas.

In our research, 40 cases of hypersensitivity reactions after G-CSF administration were documented. A total of 29 were subsequently categorized as immediate and 11 as delayed. Among the immediate reactions, filgrastim was identified as the culprit in at least 13 cases, while pegfilgrastim was implicated in most of the delayed ones with known culprits.

Although the overall incidence of hypersensitivity reactions may not be notably high, the occurrence of severe, life-threatening reactions, comprising at least 9 out of 29 cases, justifies the administration of G-CSF under careful medical supervision, allowing for immediate treatment.

Moreover, despite the conventional understanding that allergic reactions typically appear after sensitization to a drug, it is imperative to recognize the possibility of immediate hypersensitivity reactions occurring even at the initial exposure, which were present in 11 of the 29 cases (most likely not IgE-mediated), emphasizing the need for a vigilant approach in clinical assessments.

As allergy work-up is currently available, we consider it essential in the therapeutic approach of patients suffering from hypersensitivity reactions to G-CSF. Documenting the hypersensitivity reaction thoroughly is crucial for a comprehensive case analysis. Such meticulous documentation serves as a foundational step in decoding the complexities of the reaction and aids in tailoring effective management strategies.

There is still an insufficient amount of data in the literature for a meticulous analysis and numerous challenges are still posed by these hypersensitivity reactions. Despite this, even after inducing severe reactions, G-CSF is considered vital for the survival of some patients. Five desensitization protocols have been proposed and successfully applied, allowing for the safe continuation of treatment in select cases.

We believe that this study brings to light interest in this topic that could benefit from further exploration and propose regular updating to include the most recently published evidence. The need to fill the gaps in knowledge in this subject is immense, and we encourage every effort leading to it.

## Figures and Tables

**Table 2:** Non-irritating skin testing concentrations for filgrastim and lenograstim [85,105,106,107,108].

Molecule	SPT (μg/mL)	IDT (μg/mL)
Filgrastim	300	300
Lenograstim	263	263

SPT—skin prick test, IDT—intradermal test. There is the possibility of starting at a 1/1000 dilution and titrating to full strength.

**Table 3:** Current data for desensitization protocols to G-CSF molecules [93,94,95,98,99].

Author (Ref.)	Underlying Disease	Culprit	Drug for Desensitization	Route	No. of Steps	Total Duration	Time between Doses	Cumulative Dose (µg)
Gonzalez-Cavero et al. [98]	V-EST	F and L	F	iv	12	195 min	15–30 min	69.60
Jeter et al. [99]	HL	PegF	F	sc	12	180 min	15–114 min	301.05
Nunez-Acevedo et al. [94]	BC	F	L	iv	9	170 min	15–50 min	263
Hronek et al. [93]	MM	F	F	sc	15	300 min	UKN	488.33
Amaral et al. [95]	BC	F	F	iv	8	3 days	UKN	300

V-EST—vaginal endodermal sinus tumor, HL—Hodgkin’s lymphoma, BC—breast carcinoma, MM—multiple myeloma, F—filgrastim, L—lenograstim, iv—intravenous, sc—subcutaneous.

## Data Availability

Data is contained within the article.

## References

[1] Souza L., Boone T., Gabrilove J., Lai P., Zsebo K., Murdock D., Chazin V., Bruszewski J., Lu H., Chen K. (1986). Recombinant human granulocyte colony-stimulating factor: Effects on normal and leukemic myeloid cells. Science.

[2] Anderlini P. (2009). Effects and safety of granulocyte colony-stimulating factor in healthy volunteers. Curr. Opin. Hematol..

[3] Håkansson L., Höglund M., Jönsson U.B., Torsteinsdottir I., Xu X., Venge P. (1997). Effects of in vivo administration of G-CSF on neutrophil and eosinophil adhesion. Br. J. Haematol..

[4] Fraser A.R., Cook G., Franklin I.M., Templeton J.G., Campbell M., Holyoake T.L., Campbell J.D.M. (2006). Immature monocytes from G-CSF-mobilized peripheral blood stem cell collections carry surface-bound IL-10 and have the potential to modulate alloreactivity. J. Leukoc. Biol..

[5] Marino V.J., Roguin L.P. (2008). The granulocyte colony stimulating factor (G-CSF) activates Jak/STAT and MAPK pathways in a trophoblastic cell line. J. Cell. Biochem..

[6] Zhang L., Tao L., Guo L., Zhan J., Yuan C., Ma Z., Jiang B., Xiu D. (2018). G-CSF associates with neurogenesis and predicts prognosis and sensitivity to chemotherapy in pancreatic ductal adenocarcinoma. Cancer Manag. Res..

[7] Kirsch F., Krüger C., Schneider A. (2008). The receptor for Granulocyte-colony stimulating factor (G-CSF) is expressed in radial glia during development of the nervous system. BMC Dev. Biol..

[8] Vose J.M., Armitage J.O. (1995). Clinical applications of hematopoietic growth factors. J. Clin. Oncol..

[9] Lazarus H.M., Rowe J.M. (1994). Clinical use of hematopoietic growth factors in allogeneic bone marrow transplantation. Blood Rev..

[10] Pick A.M., Nystrom K.K. (2014). Nonchemotherapy drug-induced neutropenia and agranulocytosis: Could medications be the culprit?. J. Pharm. Pract..

[11] Hemmige V., Liles W.C., Pitrak D.L., Molineux G., Foote M., Arvedson T. (2011). Use of Filgrastim (r-metHuG-CSF) in Human Immunodeficiency Virus Infection. Twenty Years of G-CSF. Milestones in Drug Therapy.

[12] Smith T.J., Bohlke K., Lyman G.H., Carson K.R., Crawford J., Cross S.J., Goldberg J.M., Khatcheressian J.L., Leighl N.B., Perkins C.L. (2015). Recommendations for the use of WBC growth factors: American society of clinical oncology clinical practice guideline update. J. Clin. Oncol..

[13] Abraham I., Tharmarajah S., Macdonald K. (2013). Clinical safety of biosimilar recombinant human granulocyte colony-stimulating factors. Expert Opin. Drug Saf..

[14] Wang G., Zhang Y., Wang X., Sun Q., Xun Z., Yuan M., Li Z. (2021). Long-acting versus short-acting granulocyte colony-stimulating factors among cancer patients after chemotherapy in China. Medicine.

[15] Filon O., Nechaeva M., Burdaeva O., Vladimirov V.I., Lifirenko I., Kovalenko N.V., Kopp M.V., Matrosova M., Mukhametsina G., Panchenko S. (2015). Efficacy and safety of empegfilgrastim, a novel pegylated G-CSF: Results of complete analysis after 4 cycles of myelosuppressive chemotherapy in phase III double-dummy randomized clinical study. J. Clin. Oncol..

[16] Volovat C., Gladkov O.A., Bondarenko I.M., Barash S., Buchner A., Bias P., Adar L., Avisar N. (2014). Efficacy and safety of Balugrastim compared with pegfilgrastim in patients with breast cancer receiving chemotherapy. Clin. Breast Cancer.

[17] Farese A.M., Casey D.B., Vigneulle R.M., Siegel N.R., Finn R.F., Klover J.A., Smith W.G., McKearn J.P., MacVittie T.J. (2001). A Single Dose of Pegylated Leridistim Significantly Improves Neutrophil Recovery in Sublethally Irradiated Rhesus Macaques. Stem Cells.

[18] Cho H.W., Lee J.W., Ju H.Y., Hyun J.K., Yoo K.H., Koo H.H., Kim K., Sung K.W. (2022). Safety and Efficacy of Pegteograstim on Chemotherapy-induced Neutropenia in Children and Adolescents with Solid Tumors. J. Pediatr. Hematol. Oncol..

[19] Schriber J.R., Negrin R.S. (1993). Use and Toxicity of the Colony-Stimulating Factors. Drug Saf..

[20] Litam P.P., Friedman H.D., Loughran T.P. (1993). Splenic extramedullary hematopoiesis in a patient receiving intermittently administered granulocyte colony-stimulating factor. Ann. Intern. Med..

[21] Vidarsson B., Geirsson A.J., Önundarson P.T. (1995). Reactivation of rheumatoid arthritis and development of leukocytoclastic vasculitis in a patient receiving granulocyte colony-stimulating factor for Felty’s syndrome. Am. J. Med..

[22] Hayat S.Q., Hearth-Holmes M., Wolf R.E. (1995). Flare of arthritis with successful treatment of Felty’s syndrome with granulocyte colony stimulating factor (GCSF). Clin. Rheumatol..

[23] Yasuda M., Kihara T., Wada T., Shiokawa S., Furuta E., Suenagu Y., Nonaka S., Nobunaga M., Yoshioka K., Isayama T. (1994). Granulocyte Colony-Stimulating Factor Induction of Improved Leukocytopenia with Inflammatory Flare in a Felty’s Syndrome Patient. Arthritis Rheum..

[24] Wang W.C., Cordoba J., Infante A.J., Conley M.E. (1994). Successful treatment of neutropenia in the hyper-immunoglobulin M syndrome with granulocyte colony-stimulating factor. Am. J. Pediatr. Hematol. Oncol..

[25] Sequí-Sabater J.M., Beretta L. (2022). Defining the Role of Monocytes in Sjögren’s Syndrome. Int. J. Mol. Sci..

[26] Topcuoglu P., Arat M., Dalva K., Özcan M. (2004). Administration of granulocyte-colony-stimulating factor for allogeneic hematopoietic cell collection may induce the tissue factor-dependent pathway in healthy donors. Bone Marrow Transplant..

[27] Falanga A., Marchetti M., Evangelista V., Manarini S., Oldani E., Giovanelli S., Galbusera M., Cerletti C., Barbui T. (1999). Neutrophil activation and hemostatic changes in healthy donors receiving granulocyte colony-stimulating factor. Blood.

[28] Shimoda K., Okamura S., Inaba S., Okamura T., Ohga S., Ueda K., Niho Y. (1993). Granulocyte colony-stimulating factor and platelet aggregation. Lancet.

[29] LeBlanc R., Roy J., Demers C., Vu L., Cantin G. (1999). A prospective study of G-CSF effects on hemostasis in allogeneic blood stem cell donors. Bone Marrow Transplant..

[30] Conti J.A., Scher H.I. (1992). Acute arterial thrombosis after escalated-dose methotrexate, vinblastine, doxorubicin, and cisplatin chemotherapy with recombinant granulocyte colony-stimulating factor: A possible new recombinant granulocyte colony-stimulating factor toxicity. Cancer.

[31] Sohngen D., Wienen S., Siebler M., Boogen C., Scheid C., Schulz A., Kobbe G., Diehl V., Heyll A. (1998). Analysis of rhG-CSF-effects on platelets by in vitro bleeding test and transcranial Doppler ultrasound examination. Bone Marrow Transplant..

[32] Darie C., Boutalba S., Fichter P., Huret J.F., Jaillot P., Deplus F., Gerenton S., Zenone T., Moreau J.L., Grand A. (2004). [Aortitis after G-CSF injections]. Rev. Med. Interne.

[33] Sato Y., Kaji S., Ueda H., Tomii K. (2017). Thoracic aortitis and aortic dissection following pegfilgrastim administration. Eur. J. Cardio-Thorac. Surg..

[34] Oshima Y., Takahashi S., Tani K., Tojo A. (2019). Granulocyte colony-stimulating factor-associated aortitis in the Japanese Adverse Drug Event Report database. Cytokine.

[35] Adiga G.U., Elkadi D., Malik S.K., Fitzpatrick J.D., Madajewicz S. (2009). Abdominal aortitis after use of granulocyte colony-stimulating factor. Clin. Drug Investig..

[36] Miller E.B., Grosu R., Landau Z. (2016). Isolated abdominal aortitis following administration of granulocyte colony stimulating factor (G-CSF). Clin. Rheumatol..

[37] Taimen K., Heino S., Kohonen I., Relas H., Huovinen R., Hänninen A., Pirilä L. (2020). Granulocyte colony-stimulating factor- and chemotherapy-induced large-vessel vasculitis: Six patient cases and a systematic literature review. Rheumatol. Adv. Pract..

[38] Demuynck H., Schetz M., Berghe G.V.D., Lauwers P., Boogaerts M.A., Zache P., Verhoef G.E.G. (1995). Risks of rhG-CSF treatment in drug-induced agranulocytosis. Ann. Hematol..

[39] Laprise-Lachance M., Lemieux P., Grégoire J.P. (2019). Risk of pulmonary toxicity of bleomycin and filgrastim. J. Oncol. Pharm. Pract..

[40] van Woensel J.B.M., Knoester H., Leeuw J.A., van Aalderen W.M.C. (1994). Acute respiratory insufficiency during doxorubicin, cyclophosphamide, and G-CSF therapy. Lancet.

[41] Verhoef G., Boogaerts M. (1991). Treatment with granulocyte-macrophage colony stimulating factor and the adult respiratory distress syndrome. Am. J. Hematol..

[42] Iki S., Yoshinaga K., Ohbayashi Y., Urabe A. (1993). Cytotoxic drug-induced pneumonia and possible augmentation by G-CSF—Clinical attention. Ann. Hematol..

[43] Eisenbeis C.F., Winn D., Poelman S., Polsky C.V., Rubenstein J.H., Olopade O.I. (2001). A case of pulmonary toxicity associated with G-CSF and doxorubicin administration. Ann. Hematol..

[44] Bastion Y., Reyes F., Bosly A., Gisselbrecht C., Yver A., Gilles E., Maral J., Coiffier B. (1994). Possible toxicity with the association of G-CSF and bleomycin. Lancet.

[45] Frampton J.E., Lee C.R., Faulds D. (1994). Filgrastim: A Review of its Pharmacological Properties and Therapeutic Efficacy in Neutropenia. Drugs.

[46] Buntzel J., Kuttner K. (1995). Hepatic injury due to G-CSF application. Onkologie.

[47] Günther G., Mauz-Körholz C., Körholz D., Burdach S. (1992). G-CSF and liver toxicity in a patient with neuroblastoma. Lancet.

[48] FOSSÅ S.D., Poulsen J.P., Aaserud A. (1992). Alkaline phosphatase and lactate dehydrogenase changes during leucocytosis induced by G-CSF in testicular cancer. Lancet.

[49] Maiche A.G., Muhonen T., Porkka K. (1992). Lactate dehydrogenase changes during granulocyte colony-stimulating factor treatment. Lancet.

[50] Bonilla M.A., Dale D., Zeidler C., Last L., Reiter A., Ruggeiro M., Davis M., Koci B., Hammond W., Gillio A. (1994). Long-term safety of treatment with recombinant human granulocyte colony-stimulating factor (r-metHuG-CSF) in patients with severe congenital neutropenias. Br. J. Haematol..

[51] Minelli O., Falzetti F., Di Ianni M., Onorato M., Plebani S., Silvani C., Tabilio A. (2009). G-CSF-induced thrombocytopenia in a healthy donor. Bone Marrow Transplant..

[52] Iltar U., Salim O., Küpesiz A. (2018). Severe thrombocytopenia related to filgrastim mobilization in a healthy donor. Transfus. Apher. Sci..

[53] Vivancos J., Vila M., Serra A., Loscos J., Anguita A. (2002). Failure of G-CSF therapy in neutropenia associated with Sjögren’s syndrome. Rheumatology.

[54] Wiedl C., Walter A.W. (2010). Granulocyte colony stimulating factor in neonatal alloimmune neutropenia: A possible association with induced thrombocytopenia. Pediatr. Blood Cancer.

[55] Kango G., Haroun F. (2020). Filgrastim induced thrombocytopenia. BMJ Case Rep..

[56] Özbek N., Özyürek E. (2006). Drug-induced thrombocytopenia during G-CSF therapy in a patient with chronic neutropenia. Turk. J. Hematol..

[57] Wun T. (1993). The Felty syndrome and G-CSF-associated thrombocytopenia and severe anemia. Ann. Intern. Med..

[58] Ross H.J., Moy L.A., Kaplan R., Figlin R.A. (1991). Bullous pyoderma gangrenosum after granulocyte colony-stimulating factor treatment. Cancer.

[59] Paydas S. (2013). Sweet’s syndrome: A revisit for hematologists and oncologists. Crit. Rev. Oncol. Hematol..

[60] Park J.W., Mehrotra B., Barnett B.O., Baron A.D., Venook A.P. (1992). The Sweet syndrome during therapy with granulocyte colony-stimulating factor. Ann. Intern. Med..

[61] Park C.J., Bae Y.D., Choi J.Y., Heo P.S., Lee K.S., Park Y.S., Lee J.A. (2001). Sweet’s syndrome during the treatment of acute promyelocytic leukemia with all-trans retinoic acid. Korean J. Intern. Med..

[62] Fukutoku M., Shimizu S., Ogawa Y., Takeshita S., Masaki Y., Arai T., Hirose Y., Sugai S., Konda S., Takiguchi T. (1994). Sweet’s syndrome during therapy with granulocyte colony-stimulating factor in a patient with aplastic anaemia. Br. J. Haematol..

[63] Paydas S., Sahin B., Seyrek E., Soylu M., Gonlusen G., Acar A., Tuncer I. (1993). Sweet’s syndrome associated with G-CSF. Br. J. Haematol..

[64] Abecassis S., Ingen-Housz-Oro S., Cavelier-Balloy B., Arnulf B., Bachelez H., Dubertret L. (2004). Particularités histologiques d’un cas de syndrome de Sweet déclenché par le G-CSF. Ann. Dermatol. Venereol..

[65] van Kamp H., Van den Berg E., Timens W., Kraaijenbrink R.A., Halie M.R., Daenen S.M.G.J. (1994). Sweet’s syndrome in myeloid malignancy: A report of two cases. Br. J. Haematol..

[66] Johnson M.L. (1994). Leukocyte colony-stimulating factors. A review of associated neutrophilic dermatoses and vasculitides. Arch. Dermatol..

[67] Ferran M., Gallardo F., Salar A., Iglesias M., Barranco C., Pujol R.M. (2006). Granulomatous dermatitis with enlarged histiocytes: A characteristic pattern of granulocyte colony-stimulating factor. Report of two cases and review of the literature. Dermatology.

[68] Ostlere L.S., Harris D., Prentice H.G., Rustin M.H.A. (1992). Widespread folliculitis induced by human granulocyte-colony-stimulating factor therapy. Br. J. Dermatol..

[69] Jain K.K. (1994). Cutaneous vasculitis associated with granulocyte colony-stimulating factor. J. Am. Acad. Dermatol..

[70] Couderc L.J., Philippe B., Franck N., Balloul-Delclaux E., Lessana-Leibowitch M. (1995). Necrotizing vasculitis and exacerbation of psoriasis after granulocyte colony-stimulating factor for small cell lung carcinoma. Respir. Med..

[71] Yoon D., Byun H.J., Oh S.J., Park J.H., Lee D.Y. (2020). A case of cutaneous leukocytoclastic vasculitis associated with granulocyte colony-stimulating factor: An unusual presentation. Ann. Dermatol..

[72] Farhey Y.D., Herman J.H. (1995). Vasculitis complicating granulocyte colony stimulating factor treatment of leukopenia and infection in Felty’s syndrome. J. Rheumatol..

[73] Lee P.K., Dover J.S. (1996). Recurrent exacerbation of acne by granulocyte colony-stimulating factor administration. J. Am. Acad. Dermatol..

[74] Mössner R., Beckmann I., Hallermann C., Neumann C., Reich K. (2004). Granulocyte colony-stimulating-factor-induced psoriasiform dermatitis resembles psoriasis with regard to abnormal cytokine expression and epidermal activation. Exp. Dermatol..

[75] Upasana M., Yin W.Y., Bo W.J., Quan S.J. (2020). Granulocyte-Colony stimulating factor induced early psoriasis—A case report and literature review. Our Dermatol. Online.

[76] Yamashita N., Natsuaki M., Morita H., Kitano Y., Sagami S. (1993). Cutaneous eruptions induced by granulocyte colony-stimulating factor in two cases of acute myelogenous leukemia. J. Dermatol..

[77] Fariña M.C., Requena L., Dómine M., Soriano M.L., Estevez L., Barat A. (1998). Histopathology of cutaneous reaction to granulocyte colony-stimulating factor: Another pseudomalignancy. J. Cutan. Pathol..

[78] Crawford J., Ozer H., Stoller R., Johnson D., Lyman G., Tabbara I., Kris M., Grous J., Picozzi V., Rausch G. (1991). Reduction by Granulocyte Colony-Stimulating Factor of Fever and Neutropenia Induced by Chemotherapy in Patients with Small-Cell Lung Cancer. N. Engl. J. Med..

[79] Bustillo I., Kaley K., Saif M.W. (2009). Rash associated with the use of pegylated filgrastim in a patient with advanced pancreatic cancer. Cutan. Ocul. Toxicol..

[80] Samlaska C.P. (1993). Localized cutaneous reactions to granulocyte colony-stimulating factor. Arch. Dermatol..

[81] Cardona V., Ansotegui I.J., Ebisawa M., El-Gamal Y., Fernandez Rivas M., Fineman S., Geller M., Gonzalez-Estrada A., Greenberger P.A., Sanchez Borges M. (2020). World allergy organization anaphylaxis guidance 2020. World Allergy Organ. J..

[82] Jaiyesimi I., Giralt S.S., Wood J. (1991). Subcutaneous Granulocyte Colony-Stimulating Factor and Acute Anaphylaxis. N. Engl. J. Med..

[83] Yokoyama A., Sasaki O., Uemura S., Fujino S., Inoue Y., Kohno N., Hiwada K. (1994). Drug Eruption Caused by Recombinant Human G-CSF. Intern. Med..

[84] Batel-Copel L., Mommeja-Marin H., Oudard S., Chauvenet L., Pujade-Lauraine E., Coupier J., Bernadou A. (1995). Anaphylactic reaction after a first filgrastim (GranuLocyte-colony stimulating factor) injection. Eur. J. Cancer.

[85] Munoz R.M., Gomez-Bellver M.J., Pulido A.M.N., Cuevas J.C.O. (1996). Probable hypersensitivity reaction to filgrastim. Am. J. Health Syst. Pharm..

[86] Sullivan A.K., Nelson M.R. (1997). Allergic skin reaction to GCSF in a patient with AIDS. Int. J. STD AIDS.

[87] Adkins D.R. (1998). Anaphylactoid reaction in a normal donor given granulocyte colony-stimulating factor. J. Clin. Oncol..

[88] Keung Y.K., Suwanvecho S., Cobos E. (1999). Anaphylactoid reaction to granulocyte colony-stimulating factor used in mobilization of peripheral blood stem cell. Bone Marrow Transplant..

[89] Khoury H., Adkins D., Brown R., Vij R., Westervelt P., Trinkaus K., Goodnough L.T., DiPersio J.F. (2000). Adverse side-effects associated with G-CSF in patients with chronic myeloid leukemia undergoing allogeneic peripheral blood stem cell transplantation. Bone Marrow Transplant..

[90] Hanna G.G., Edgar D., Clarke J.E.M. (2008). A Case of Prolonged Type 1 Hypersensitivity Reaction to Pegfilgrastim. Clin. Oncol..

[91] Tulpule S., Shaw B.E., Makoni P., Little A.M., Madrigal J.A., Goldman J.M. (2009). Severe allergic reaction with anaphylaxis to G-CSF (lenograstim) in a healthy donor. Bone Marrow Transplant..

[92] Tholpady A., Chiosea I., Lyons J.J., Baird K., Leitman S.F. (2013). Systemic hypersensitivity reaction mimicking anaphylaxis after first filgrastim administration in a healthy donor. Transfusion.

[93] Hronek B., Kulczycki A. (2014). A Successful Desensitization Protocol For Filgrastim. J. Allergy Clin. Immunol..

[94] Núñez-Acevedo B., Rodríguez-Jiménez B., Domínguez-Ortega J., González-Montellano E., Ibáñez-Heras N., Enrech-Francés S. (2015). Desensitization to lenograstim after a lifethreatening reaction to filgrastim. J. Investig. Allergol. Clin. Immunol..

[95] Carr D.F., Chung W.-H., Jenkiins R.E., Chaponda M., Nwikue G., Cornejo Castro E.M., Antoine D.J., Pirmohamed M., Wuillemin N., Dina D. (2016). 7th drug hypersensitivity meeting: Part one. Clin. Transl. Allergy.

[96] Yamamoto K., Doki N., Senoo Y., Najima Y., Kobayashi T., Kakihana K., Haraguchi K., Okuyama Y., Sakamaki H., Ohashi K. (2016). Severe Hypoxemia in a Healthy Donor for Allogeneic Hematopoietic Stem Cell Transplantation after only the First Administration of Granulocyte-Colony Stimulating Factor. Transfus. Med. Hemotherapy.

[97] Doval D., Choudhary D., Sharma S.K., Khandelwal V. (2019). Severe hypersensitivity allergic reaction to filgrastim in a healthy stem cell donor. J. Oncol. Pharm. Pract..

[98] González-Cavero L., Gómez-Traseira C., Fiandor A., Entrala A., Quirce S. (2019). Desensitization to filgrastim in a 2-year-old girl with a vaginal endodermal sinus tumor. J. Investig. Allergol. Clin. Immunol..

[99] Jeter H., Grisanti K., Jantz A., Wadhwa A., Waite E. (2022). Filgrastim desensitization in a patient with Hodgkin lymphoma. Pediatr. Blood Cancer.

[100] Álvarez Ruiz S., Fernández-Peñas P., Sánchez Pérez J., Fernández-Herrera J., García Díez A., Fraga Fernándeza J. (2003). Erupción por factores estimuladores de colonias granulocíticas en un paciente con leucemia mieloide crónica. Actas Dermosifiliogr..

[101] Álvarez-Ruiz S., Peñas P.F., Fernández-Herrera J., Sánchez-Pérez J., Fraga J., García-Díez A. (2004). Maculopapular eruption with enlarged macrophages in eight patients receiving G-CSF or GM-CSF. J. Eur. Acad. Dermatol. Venereol..

[102] Brumit M.C., Shea T.C., Brecher M.E. (2003). G-CSF-associated rash in an allogeneic PBPC donor. Transfusion.

[103] Scott W.R., Silberstein L., Flatley R., Ardeshna K.M., Korostoff N., Dawe S. (2009). Cutaneous reaction to pegfilgrastim presenting as severe generalized skin eruption. Br. J. Dermatol..

[104] Dadla A., Tannenbaum S., Yates B., Holle L. (2015). Delayed hypersensitivity reaction related to the use of pegfilgrastim. J. Oncol. Pharm. Pract..

[105] Stone H.D., Dipiro C., Charlton Davis P., Meyer C.F., Wray B.B. (1998). Hypersensitivity reactions to Escherichia coli-derived polyethylene glycolated-asparaginase associated with subsequent immediate skin test reactivity to E. coli-derived granulocyte colony-stimulating factor. J. Allergy Clin. Immunol..

[106] Shelley W.B., Talanin N., Shelley E.D. (1995). Polysorbate 80 hypersensitivity. Lancet.

[107] Levy M., Dupuis L.L. (1990). Parenteral Nutrition Hypersensitivity. J. Parenter. Enter. Nutr..

[108] Engler R.J.M., Weiss R.B. (1996). Immediate hypersensitivity to human recombinant granulocyte-macrophage colony-stimulating factor associated with a positive prick skin test reaction. Ann. Allergy Asthma Immunol..

